# Experimental and Numerical Investigation of Polymer-Based 3D-Printed Lattice Structures with Largely Tunable Mechanical Properties Based on Triply Periodic Minimal Surface

**DOI:** 10.3390/polym16050711

**Published:** 2024-03-05

**Authors:** Zhenjie Wang, Menghui Xu, Jianke Du, Yuan Jin

**Affiliations:** 1 Smart Materials and Advanced Structure Laboratory, School of Mechanical Engineering and Mechanics, Ningbo University, Ningbo 315211, China; 2 Science and Technology Department, Xinjiang Institute of Technology, Akesu 843100, China

**Keywords:** lattice structures, anisotropy, mechanical properties, energy absorption capability, finite element analysis

## Abstract

Triply periodic minimal surfaces (TPMSs) have demonstrated significant potential in lattice structure design and have been successfully applied across multiple industrial fields. In this work, a novel lattice structure with tunable anisotropic properties is proposed based on two typical TPMS types, and their mechanical performances are studied both experimentally and numerically after being fabricated using a polymer 3D printing process. Initially, adjustments are made to the original TPMS lattice structures to obtain honeycomb lattice structures, which are found to possess significant anisotropy, by utilizing numerical homogenization methods. Based on this, a continuous self-twisting deformation is proposed to change the topology of the honeycomb lattice structures to largely tune the mechanical properties. Quasi-static compression experiments are conducted with different twisting angles, and the results indicate that self-twisting can affect the mechanical properties in specific directions of the structure, and also enhance the energy absorption capacity. Additionally, it mitigates the risk of structural collapse and failure during compression while diminishing structural anisotropy. The proposed self-twisting strategy, based on honeycomb lattice structures, has been proven valuable in advancing the investigation of lattice structures with largely tunable mechanical properties.

## 1. Introduction

Additive manufacturing (AM) has revolutionized the manufacturing industry by enabling the production of complex and customized objects with unprecedented precision via building objects layer by layer using a variety of materials, including polymers, ceramics, and metals [1]. One remarkable application of AM is the creation of lattice structures, which mimic the intricate architecture found in nature [2,3,4]. These structures are characterized by a lattice-like pattern of interconnected struts or beams, resulting in a lightweight yet robust design. By leveraging the unique capabilities of additive manufacturing, engineers and designers can fabricate lattice structures with precise control over their geometry, density, and material composition [5,6]. Lattice structures have been applied across various fields by providing structural integrity while minimizing material usage, as well as enabling efficient heat and fluid transfer [7,8].

Since AM allows for the creation of complex internal architectures that are otherwise impossible to manufacture using traditional methods, lattice structures can be optimized to exhibit specific mechanical properties, such as enhanced stiffness, energy absorption, or flexibility, by adjusting the geometry and orientation of the unit cells [9,10,11]. Various lattice topologies, such as diamond, cubic, and octet, have been developed using advanced computational techniques and considering engineering principles [12]. Through an iterative design process and leveraging the unique capabilities of AM, lattice structures can be tailored to meet specific performance requirements, resulting in highly efficient and customized structures for a wide range of applications.

A triply periodic minimal surface (TPMS) is a mathematical concept that describes smooth surfaces with zero curvatures in three mutually orthogonal directions [13]. TPMS structures exhibit a uniform distribution of stresses, providing enhanced load-bearing capabilities and improved structural integrity. By combining the geometric characteristics of a TPMS with the design freedom offered by AM, it is possible to create innovative and efficient structures that were previously unattainable using traditional manufacturing methods. Thanks to the advent of AM, TPMS lattice structures offer unique advantages, such as lightweight, strong, and highly porous structures with optimized mechanical properties; thus, applications in various fields can be found, including in aerospace [14], automotive [15], and biomedical industries [16].

The mechanical properties of TPMS lattice structures have been comprehensively studied to understand their unique behavior and to build the relationship between the geometric parameters of TPMS lattice structures, such as cell size, strut thickness, and porosity, and their mechanical properties, including stiffness, strength, and energy absorption capacity [17]. In particular, TPMS lattice structures have emerged as highly effective solutions for enhancing energy absorption systems across various applications. Their intricate geometric arrangement and robust mechanical properties make them indispensable in mitigating dynamic loading events and minimizing damage. From automotive safety features to sports equipment design, TPMS lattice structures offer unparalleled performance in dissipating energy, thereby significantly improving overall safety standards. The quasi-static [18,19,20] and dynamic mechanical performance [21,22,23] of TPMS lattice structures have been comprehensively investigated experimentally and numerically. Through experimental testing and numerical simulations, studies have aimed to characterize the load-bearing capacity, deformation behavior, and failure mechanisms of TPMS-based lattice structures under different loading conditions.

Besides the common mechanical properties, anisotropy is an important consideration in the design and performance evaluation of lattice structures [24]. The anisotropic behavior of lattice structures is influenced by various factors, including build orientation, scanning strategies, and processing parameters [25,26]. Experimental testing, such as tensile, compression, and bending tests, can be conducted to measure mechanical properties along different directions and evaluate the structural response under varying loading conditions. Advanced imaging techniques, such as X-ray computed tomography (CT), have been employed to analyze the microstructure and internal features of lattice structures and identify any directional variations [27,28,29]. Numerical simulations, including finite element analysis (FEA), have also been utilized to model and predict the anisotropic behavior of lattice structures [30].

The anisotropic behavior of additively fabricated lattice structures introduces limitations and challenges in terms of mechanical properties, design flexibility, analysis, material utilization, and integration. These disadvantages must be carefully addressed and mitigated to fully leverage the benefits of lattice structures in practical applications. However, the anisotropic behavior of lattice structures can also present certain advantages and specific applications [31]. For example, anisotropic lattice structures allow for the customization of mechanical properties in different directions. This can be advantageous in applications where specific load-bearing characteristics are required, such as in aerospace components or structural supports. By optimizing the anisotropic behavior, it is possible to design structures that are stronger and stiffer along certain axes while providing the desired flexibility or compliance in other directions. At the same time, less material is needed compared to isotropic structures, resulting in material savings and reduced weight by designing the structure to have higher mechanical properties in the directions where the load is expected. Moreover, the anisotropic behavior of lattice structures can also be leveraged to integrate additional functionalities or features. By strategically designing the orientation-dependent properties, it becomes possible to embed channels for fluid flow, sensors, electrical pathways, or heat dissipation. This enables the creation of multifunctional components that combine structural support with other desired functionalities. Overall, it is important to note that the advantages and applications of anisotropic lattice structures heavily depend on the specific requirements and constraints of the given application. Careful consideration and analysis are necessary to harness the benefits of anisotropic behavior while mitigating its potential drawbacks.

Some studies have been conducted on the anisotropic behavior of TPMS lattice structures. Feng et al. [32] designed an isotropic TPMS structure and studied the relationship between the parameters of the TPMS and its anisotropic properties. On this basis, an adjustment method is proposed to control the anisotropy. Qiu et al. [33] proposed a new mixed gradient TPMS structure based on the geometric deformation gradient and volume fraction gradient. Compared with the uniform structure, the two mixed gradient structures can greatly improve the energy absorption capacity. Chatzigeorgiou et al. [34] proposed a comprehensive method to systematically study and compare the elastic properties and stiffness anisotropy of various TPMS-based and pillar-based lattices. Chen et al. [35] used the homogenization method based on FFT to study the mechanical properties of honeycomb materials with a TPMS topology. Khaleghi et al. [30] studied the directional elastic modulus of seven different TPMS structures. The results show that the stiffness tensors of all structures are almost cubic symmetrical and show the considerable directional dependence of elastic modulus. In addition, their team also found that the hybrid structure can effectively reduce anisotropy. According to the homogenization method, Li et al. [36] evaluated the effective stiffness matrix of a gyroid-based lattice structure and analyzed its elastic anisotropy. The results show that the elastic anisotropy of the lattice structure is highly dependent on the volume fraction, and the Young’s modulus of the gyroid lattice varies slightly in different directions. Through numerical modeling and compression tests, Ashcroft et al. [37] studied three kinds of gyroid-based lattice structures and obtained their anisotropy information. In addition, it was also found that the volume fraction is a particularly sensitive parameter for the anisotropy of the new honeycomb structures. Lu et al. [25] used a numerical homogenization method and analytical method to study the anisotropic elastic behavior of five commonly used TPMS stents.

This work proposes a new method to adjust the anisotropy and enhance the controllability of the mechanical properties of TPMS-based lattice structures, taking into account the specific requirements of certain applications and the characteristics of TPMSs. The method involves the choice of cross-sections, stretching, and twisting of TPMS lattice structures. By modifying the cross-sectional shape, the stress distribution in different directions can be influenced, thereby adjusting the mechanical properties of the structures in those specific directions. Additionally, deformation methods such as stretching and twisting can be employed to alter the internal details and connectivity of the TPMS structure, further tuning its mechanical properties. This approach offers a flexible way to design TPMS lattice structures to achieve specific mechanical characteristics to meet the performance requirements of targeted applications. This research opens up new possibilities for expanding the design space and application areas of TPMS lattice structures.

## 2. Materials and Methods

### 2.1. Topological Design

TPMS-based lattice structures have been widely investigated and several TPMS types have been proposed to construct lattice structures. Common TPMS-based lattice structures typically possess similar mechanical properties in three orthogonal directions due to spatial geometric symmetry, whereas honeycomb structures designed based on TPMSs can exhibit different mechanical properties in various directions [38]. To further regulate and control the mechanical properties, the TPMS-based honeycomb structure is further deformed by self-twisting in this work, and the mechanical properties of the twisted honeycomb TPMS structures along different directions are comprehensively studied.

This work focuses on both the primitive and gyroid types, and it should be noted that the proposed method can be easily adaptable to other types of TPMS-based lattice structures. The mathematical expressions of the primitive and gyroid surfaces are as follows:

(1) $$U_{P}=cos\left(\frac{2\pi x}{Lx}\right)+cos\left(\frac{2\pi y}{Ly}\right)+cos(\frac{2\pi z}{Lz})-t$$

(2) $$U_{G}=sin\left(\frac{2\pi x}{Lx}\right)cos\left(\frac{2\pi y}{Ly}\right)+sin\left(\frac{2\pi z}{Lz}\right)cos\left(\frac{2\pi x}{Lx}\right)+sin\left(\frac{2\pi y}{Ly}\right)cos(\frac{2\pi z}{Lz})-t$$

where *Lx*, *Ly*, and *Lz* are the coefficients that control the cell size in the *x*, *y*, and *z* directions, respectively, and *t* is a specific constant used to define the surface location.

To generate a honeycomb surface, one of the coordinate parameters (*x*, *y*, *z*) must be fixed at a certain value. Based on our previous study [39], different values lead to distinct cross-sectional profiles, consequently yielding diverse honeycomb structures. To secure a structural configuration that exhibits excellent mechanical performance, the parameters are designated as one-fourth of the periodic length for *U_p_* and one-eighth of the periodic length for *U_G_*, respectively. With these specifications, the mathematical equation transforms into the following form when *y* is set as *Ly*/4 and *Ly*/8, respectively.

(3) $$U_{P\_honey}=cos\left(\frac{2\pi x}{Lx}\right)+cos\left(\frac{\pi }{2}\right)+cos(\frac{2\pi z}{Lz})-t$$

(4) $$U_{G\_honey}=sin\left(\frac{2\pi x}{Lx}\right)cos\left(\frac{\pi }{2}\right)+sin\left(\frac{2\pi z}{Lz}\right)cos\left(\frac{2\pi x}{Lx}\right)+sin\left(\frac{\pi }{2}\right)cos(\frac{2\pi z}{Lz})-t$$

Using the generated honeycomb surface as a base, the honeycomb-based lattice surface can undergo self-twisting to alter its cross-sections along a specific axis. From a geometric perspective, this involves rotating coordinates around the specific axis. For illustration, when the surface is twisted around the *z*-axis, the coordinate transformation can be expressed as follows:

(5) $$\left\{\begin{array}{c} x=\sqrt{x_{0}^{2}+y_{0}^{2}}*cos[atan2\left(y_{o},x_{o}\right)+A*\frac{\left(z_{o}-z_{min}\right)}{z_{max}-z_{min}}]\\ y=\sqrt{x_{0}^{2}+y_{0}^{2}}*sin[atan2\left(y_{o},x_{o}\right)+A*\frac{\left(z_{o}-z_{min}\right)}{z_{max}-z_{min}}]\\ z=z_{o} \end{array}\right.$$

where *A* is the rotation angle of the entire lattice structure, and *z_max_* and *z_min_* represent the maximum and minimum *z* coordinate of the structure, respectively. (*x_o_*, *y_o_*, *z_o_*) and (*x*, *y*, *z*) are the original and the updated coordinates. By combining Equations (4) and (5), a honeycomb surface with self-twisted characteristics can be derived. The honeycomb-based lattice structures can be subsequently obtained by thickening the surface.

**Figure 1:**
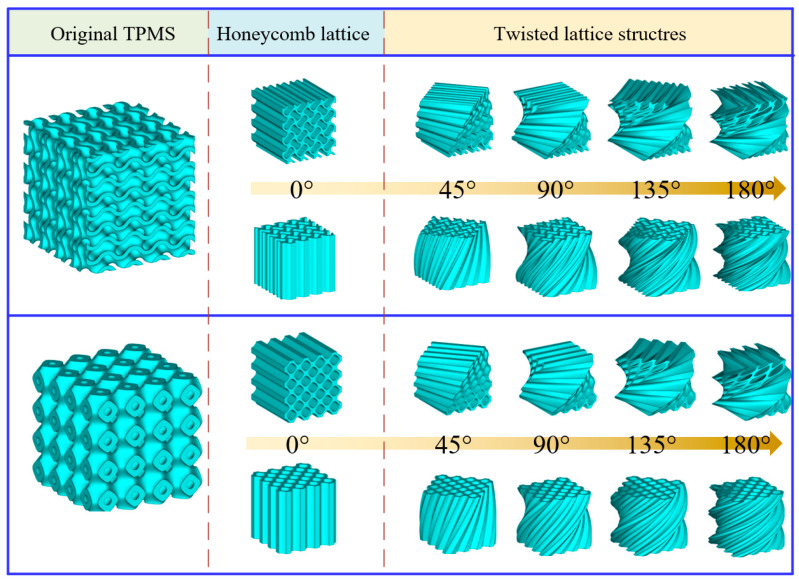
Schematic diagram of the generation of a honeycomb-based lattice structure with self-twisted characteristics.

As illustrated in Figure 1, four different twisting angles, namely 45°, 90°, 135°, and 180°, are chosen for investigation, respectively. For each type, the honeycomb structures are generated using two distinct directions (*y* and *z*). The external dimensions of the original lattice structures are 40 mm × 40 mm × 40 mm. The wall thicknesses of both the primitive and gyroid lattice structures are approximately 1.1 mm, and the cross-sectional areas for both types are nearly identical. All the generated twisted structures for each type maintain uniform cross-sections compared to the original lattice structures at different specified heights, and their total heights are identical. Consequently, based on the principles of integration, these structures have equivalent volumes. This uniformity in cross-sectional areas and height ensures that, despite any differences in their external geometries, their volumetric properties remain consistent. Based on the design model, the theoretical relative densities of all the lattice structures are 37.7%.

### 2.2. Specimen Preparation and Experimental Tests

**Figure 2:**
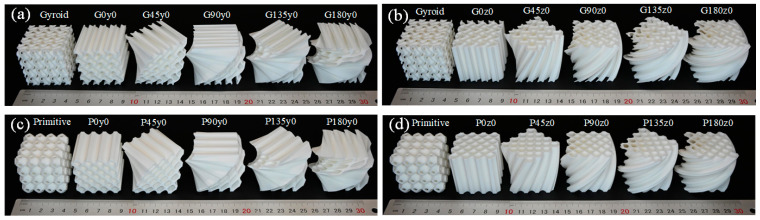
Fabricated samples with four different twisting angles and two different directions. (**a**) gyroid-type samples with fixed *y* coordinate; (**b**) gyroid-type samples with fixed *z* coordinate; (**c**) primitive-type samples with fixed *y* coordinate; (**d**) primitive -type samples with fixed *z* coordinate.

All the samples used in this study were manufactured by the selective laser sintering (SLS) process using polyamide PA2200 (EOS GmbH, Munich, Germany) as the material. The EOS P396 laser sintering machine (EOS GmbH, Munich, Germany) was adopted, equipped with a 70 W carbon dioxide laser, with a maximum output power of 10 kw. The diameter of the focused beam was 0.3 mm. All the printed samples are shown in Figure 2. For convenience of description, G0y0 indicates that the *y* coordinate of the gyroid-based honeycomb structure is fixed, with a twisting angle of 0°. Similarly, G45z0 indicates that the *z* coordinate is fixed, with a twisting angle of 45°. Primitive-based lattice structures are named in a similar naming convention. To verify the printing quality, all the samples were weighted, and the deviations between samples for each design were found to be smaller than 0.3 g. Meanwhile, the measured relative densities were all quite consistent with the theoretical values. These results confirm the reliability and consistency of the printing process for the designed lattice structures.

The quasi-static compression test of the sample was carried out by using a universal testing machine (MTS810, Eden Prairie, MN, USA, the maximum load capacity is 50 kN). In order to ensure the reliability of the test, at least three samples were made and tested for each design. The sample was compressed at a speed of 2 mm/min, and the force and displacement data were recorded during the experiment. When the displacement reaches a certain value (32 mm, where the strain is 0.8) or the force reaches the set value (48 kN), the experiment stops. The stress is obtained by dividing the force values by the cross-sectional area of the specimen, while the strain is calculated by dividing the displacement values by the original length of the specimen (40 mm). The Young’s modulus, yield stress, and energy absorption are all determined based on the obtained stress–strain curves. Meanwhile, a camera was used to record the whole process of the experiment in order to observe the deformation of the sample in the compression process.

### 2.3. Numerical Studies

**Figure 3:**
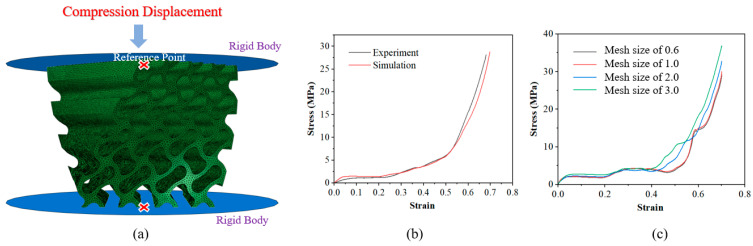
Demonstration of numerical studies: (**a**) FEA model of G45y0; (**b**) experimental and simulation results of G45y0; and (**c**) mesh sensitivity analysis.

A numerical study was carried out by using the commercial finite element (FE) software Abaqus (version 6.14-4) to understand how the twisting characteristic influences the mechanical properties of the structure in different directions. The finite element model is shown in Figure 3a. The improved quadratic tetrahedral element (C3D10M) was used in the model, and the model was sandwiched between two rigid plates to establish automatic surface contact (friction coefficient is 0.2). To ensure the convergence and reliability of the FE model, a mesh sensitivity analysis was conducted by repeatedly reducing the mesh size and obtaining the strain–stress curve until changes in the results were negligible. Using this method, the value of 0.6 is determined for the mesh size and the number of meshes for lattice structures ranges from about 0.67 to 1.11 million. During the process of compression, the upper steel plate is fixed, and the lower steel plate moves uniformly from the bottom to the top. The specific parameters used were based on previous research [37,40]. Figure 3b shows the comparison of the experimental and simulation results of the G45y0 lattice structure. It can be seen from the diagram that the two curves fit closely, indicating that the simulation results are quite reliable.

**Figure 4:**
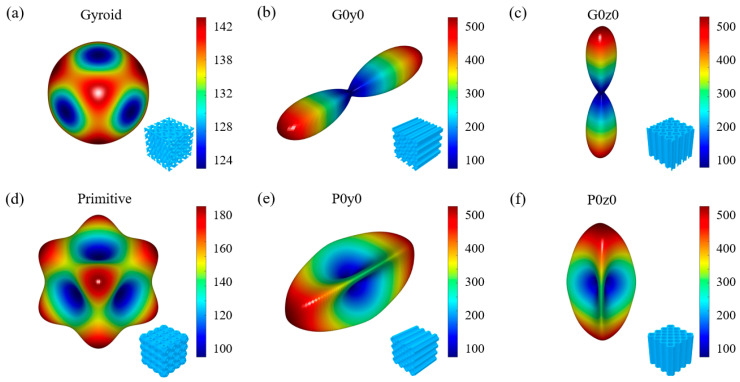
Homogenized relative elastic moduli for the gyroid and primitive types, and their honeycomb lattice structures.

Moreover, to assess the stiffness anisotropy of the lattice structures, the 3D finite element homogenization method proposed by Dong et al. [41] is employed. With this approach, the code can be easily utilized to compute the homogeneous stiffness matrix of the cell, and subsequently, the 3D modulus surface of the cell can be generated. Representative surfaces for the gyroid and primitive structures and their respective honeycomb lattices are illustrated in Figure 4. The figure reveals that the modulus surface of the gyroid structure is spherical, indicating high isotropy. Conversely, the primitive structure exhibits a larger modulus at the vertex. Notably, their honeycomb structures exhibit conspicuous anisotropy. Take G0y0 as an example; because G0y0 stretches the gyroid structure in the y direction, it has a larger modulus in the y direction. This is consistent with the performance in Figure 4b. Similarly, G0z0 stretches the gyroid structure in the z direction, so it has a larger modulus in the z direction. This is consistent with the performance in Figure 4c. This phenomenon also exists in the primitive honeycomb structure.

## 3. Results and Discussion

### 3.1. Experimental Results

The compression experiments are divided into four groups: Gy0, Gz0, Py0, and Pz0. The experimental results showed a high degree of consistency across three specimens of each design; thus, data from only one specimen per design were selected for analysis.

**Figure 5:**
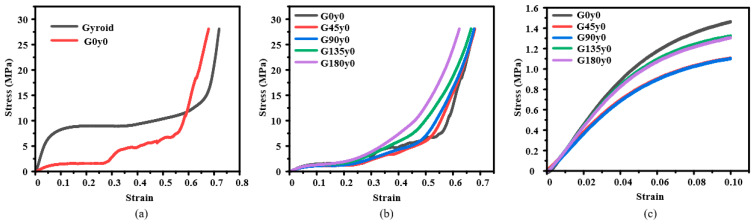
Experimental results of Gy0: (**a**) honeycomb lattice structures with and without twisting; (**b**) structures with different twisting angles; and (**c**) stress–strain curves during elastic stage from (**b**).

When the *y* coordinate in the mathematical expression of the gyroid structure is set to a fixed value, the stress–strain curve of Gy0 is obtained by quasi-static compression along the *z*-axis, as shown in Figure 5. The stress–strain curves of G and G0y0 are shown in Figure 5a. It can be seen from Figure 5a that after setting the *y* coordinate of the gyroid structure to a fixed value, the Young’s modulus of the structure decreases greatly in the elastic stage, and the stress peak value in the elastic stage decreases greatly and enters the platform stage when the strain is about 0.1. After that, the stress of the structure increases suddenly when the strain is about 0.27, then increases slowly with the increase in the strain, and finally enters the densification stage when the strain is about 0.55. It can be seen from Figure 5b that the platform stage of the structure shortens with the increase in the twisting angle. Figure 5c shows the corresponding stress–strain curve when the strain is less than 0.1. The diagram indicates that twisting slightly reduces the Young’s modulus of the structure; however, as the angle increases, the Young’s modulus does not significantly change.

**Figure 6:**
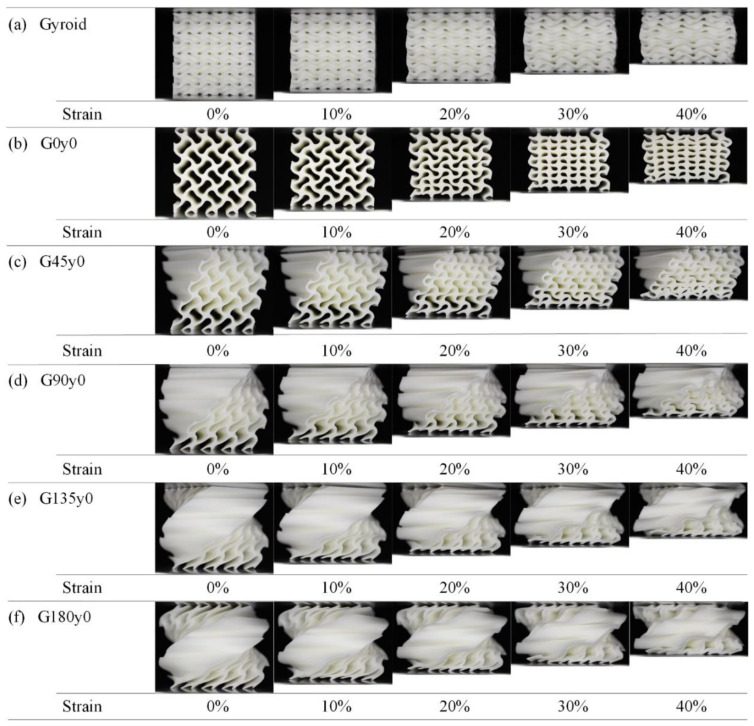
Deformation process of Gy0 lattice structures with strain from 0 to 40%.

Figure 6 shows the deformation of lattice structures in the first group with strains ranging from 0 to 40% during the quasi-static compression experiments. With regard to the gyroid structure, it can be seen from Figure 6a that in the process of compression, the deformation is very uniform, there is no stress concentration in some areas, and there is no obvious fracture, which is consistent with the stress–strain curve. Figure 6b shows the G0y0 structure. During the compression process of G0y0, the deformation of the structure as a whole is relatively uniform, and the fracture mainly occurs in the corner; the deformation of the upper right corner is different, and the fracture occurs outwardly, resulting in an obvious stress concentration. This may be attributed to differences in the element structure of the lower right corner and lower left corner. Figure 6c–f show that the deformation of the deformed Gy0 structure is more uniform during the compression process, and the plastic deformation of the structure is buffered by the twisting behavior. This phenomenon is also reflected in the stress–strain curve, where those of the twisting structures appear smoother.

**Figure 7:**
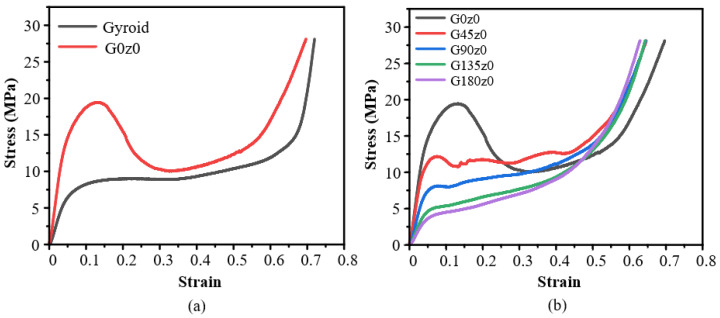
Experimental results of Gz0: (**a**) honeycomb lattice structures with and without twisting; (**b**) structures with different twisting angles.

When the *z* coordinate in the mathematical expression of the gyroid structure is set to a fixed value, the stress–strain curve of Gz0 is also obtained by quasi-static compression along the *z*-axis, as shown in Figure 7. Figure 7a shows the stress–strain curves of the gyroid and G0z0 structures. It can be seen that compared with the gyroid structure, G0z0 has a larger Young’s modulus in the elastic stage and a much higher peak stress. When the strain is about 0.12, the stress drops suddenly and the structure collapses plastically. When the strain is about 0.3, the deformation process enters the platform stage. Finally, when the strain is about 0.5, the structure enters the compaction stage. Along this direction, the stress of G0z0 is greater than that of the gyroid structure, and the bearing capacity is stronger. Figure 7b shows the stress–strain curves of Gz0 with different twisting angles. It can be seen that with the increase in the twisting angles, the Young’s modulus of the structure decreases, the degree of plastic collapse decreases or even disappears, and the structure strength under load gradually decreases, leading to a quick entry into the densification stage.

**Figure 8:**
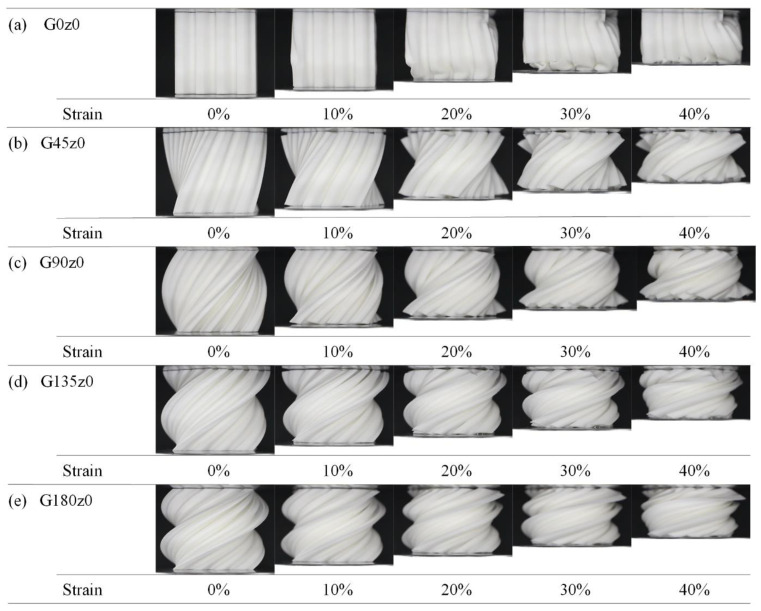
Deformation process of Gz0 lattice structures with strain from 0 to 40%.

Figure 8 shows the deformation of the structures in the second group with strains ranging from 0 to 40% during the quasi-static compression experiments. The obvious instability of G0z0 during compression is mainly manifested as tilting to one side, which leads to an obvious stress concentration on the left side and the upper right side, and finally leads to excessive bending and fracture. It can be seen from Figure 8b–e that the self-twisting behavior buffers the plastic deformation of the structure. With the increase in the twisting angle, the instability disappears, and the bearing capacity becomes more stable. This is consistent with the change in the stress–strain curves.

**Figure 9:**
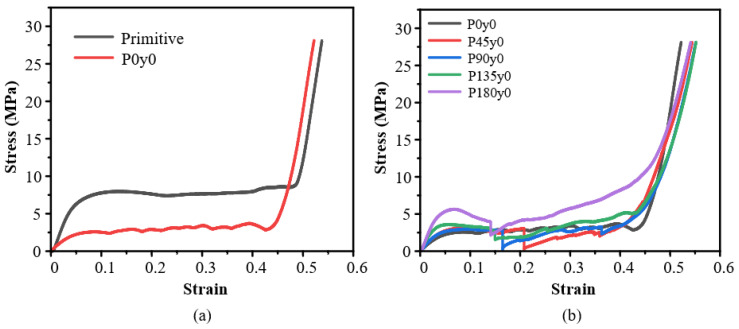
Experimental results of Py0: (**a**) honeycomb lattice structures with and without twisting; (**b**) structures with different twisting angles.

When the *y* coordinate in the mathematical expression of the primitive structure is set to a fixed value, the stress–strain curve of Py0 is obtained by quasi-static compression along the *z*-axis, as shown in Figure 9. As can be seen from Figure 9a, compared with the primitive structure, the Young’s modulus of P0y0 in the elastic stage decreases obviously, and the peak stress in the elastic stage also decreases obviously, and enters the platform stage and compaction stage more quickly, which is similar to the situation of the gyroid and G0y0 structures in the first group, except that there is no sudden increase in the stress of G0y0 in P0y0. Although there are small fluctuations up and down, it should be the intermittent destruction of the structure in the process of being compressed. Figure 9b reflects the change in the mechanical properties of the Py0 structure as the twisting angle increases. It can be seen that with the increase in the angle, the Young’s modulus in the elastic stage of the structure increases gradually, especially for 135° and 180°. In the middle platform stage, the self-twisted structure is destroyed, and the failure of P45y0 occurs the latest when the strain is 0.21. With the increase in the angle, the time of damage is gradually advanced, and the degree of damage is gradually reduced.

**Figure 10:**
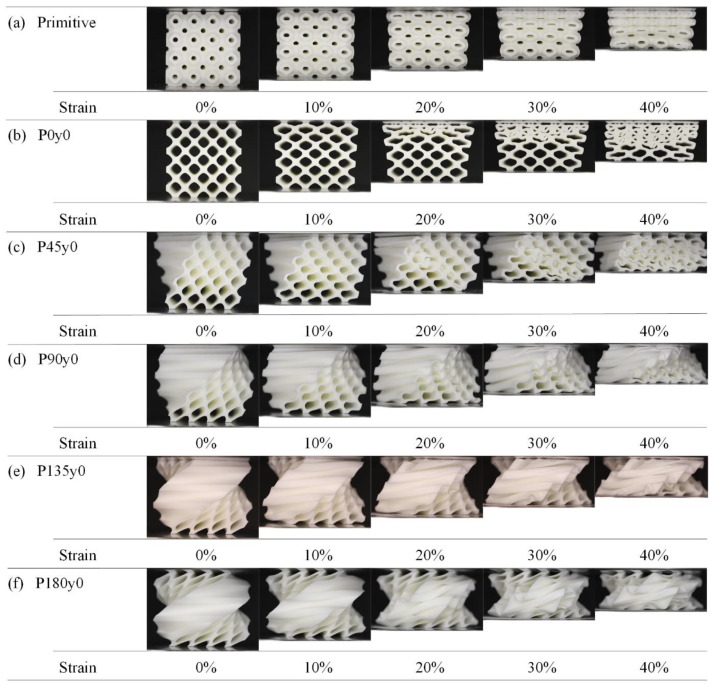
Deformation process of Py0 lattice structures with strain from 0 to 40%.

Figure 10 shows the deformation of the third group with strains ranging from 0 to 40% during the quasi-static compression experiments. As can be seen from Figure 10a,b, unlike the previous two groups, the deformation of the primitive type and its honeycomb structures starts from the top, and the elements in the first and second rows deform first and then progress downwards. However, this pattern changes once the situation is reversed. Figure 10c shows the deformation of the P45y0 structure. It is evident that the structure’s damage initiates mainly along the diagonal and then gradually spreads to both sides. With the further increase in the twisting angle, the damage degree of the structure decreases gradually.

**Figure 11:**
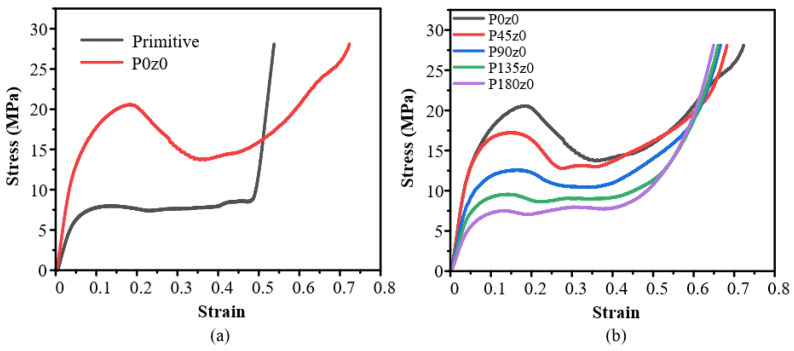
Experimental results of Pz0: (**a**) honeycomb lattice structures with and without twisting; (**b**) structures with different twisting angles.

When the *z* coordinate in the mathematical expression of the primitive structure is set to a fixed value, the stress–strain curve of Pz0 is obtained by quasi-static compression along the *z*-axis, as shown in Figure 11. It can be seen from Figure 11a that the Young’s modulus of P0z0 is larger than that of the primitive structure in the elastic stage, and the stress peak value is higher. When the strain is about 0.17, plastic failure occurs and the stress decreases. When the strain is about 0.35, the stress drop stops and enters the densification stage only after a very short platform period. Figure 11b shows the stress and strain of Pz0 structures with different angles. It can be seen that with the increase in the twisting angle, the Young’s modulus of the structure in the elastic stage decreases gradually, the peak stress decreases gradually, the plastic collapse gradually weakens, and the platform period becomes longer. The increase in the angle reduces the degree of plastic collapse of the structure in the compression process.

**Figure 12:**
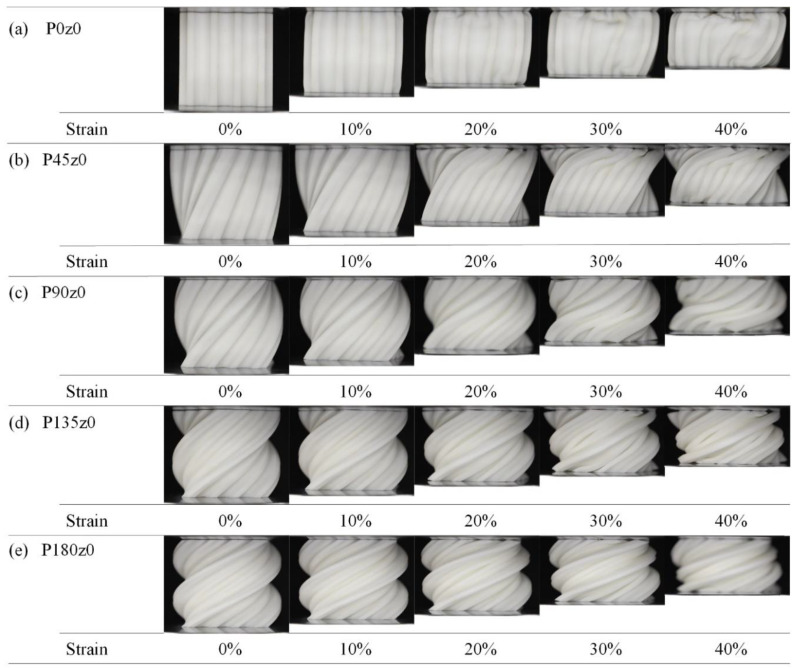
Deformation process of Pz0 lattice structures with strain from 0 to 40%.

Figure 12 shows the deformation of the fourth group with strains ranging from 0 to 40% during the quasi-static compression experiments. As can be seen from Figure 12a, the deformation of P0z0 starts from the upper and lower sides. With the increase in the strain, the structure gradually expands outward, and the deformation of the structure begins to tilt to the right. The deformation of the rightmost element is particularly obvious, showing instability similar to that observed in G0z0. It can be seen from Figure 12b–f that after being self-twisted, the structure instability disappears, the degree of plastic collapse weakens, and the overall stability improves, which is consistent with the stress–strain curve in Figure 12. Simultaneously, its performance is similar to that observed in the Gz0 group.

### 3.2. Numerical Results

**Figure 13:**
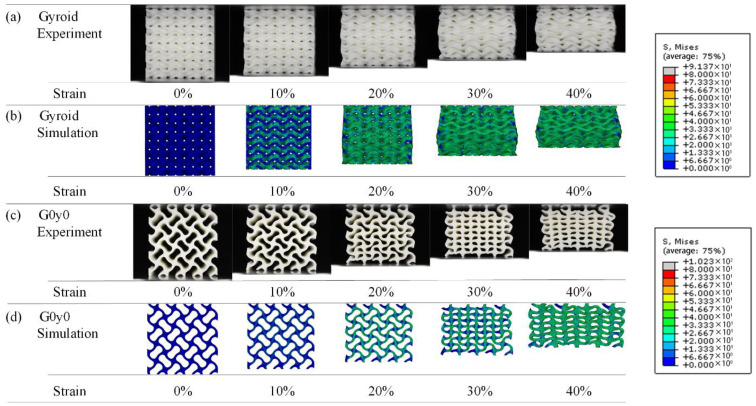
Comparison between experimental and numerical results of gyroid and G0y0 structures.

To better understand the deformation mechanism of lattice structures with different twisting angles under quasi-static compression, a finite element simulation analysis for each group of structures was conducted. Images under different strains are captured and compared with those obtained from the experimental process. Taking Gy0 as an example, Figure 13 shows the comparison between experimental results and simulation results of the gyroid and G0y0 structures in the strain range of 0–40%. From Figure 13a,b, it can be seen that the deformation of the gyroid structure in the simulation is consistent with that in the experiment, the overall stress is more uniform, and there is no obvious stress concentration. It can be seen from Figure 13c,d that the deformation of G0y0 in the simulation is consistent with that in the experiment. Moreover, it can be seen from the simulation results that the stress in the area where the elements are connected is larger. This may be attributed to bending phenomena in the connected areas, likely causing bending deformation and more concentrated stress.

**Figure 14:**
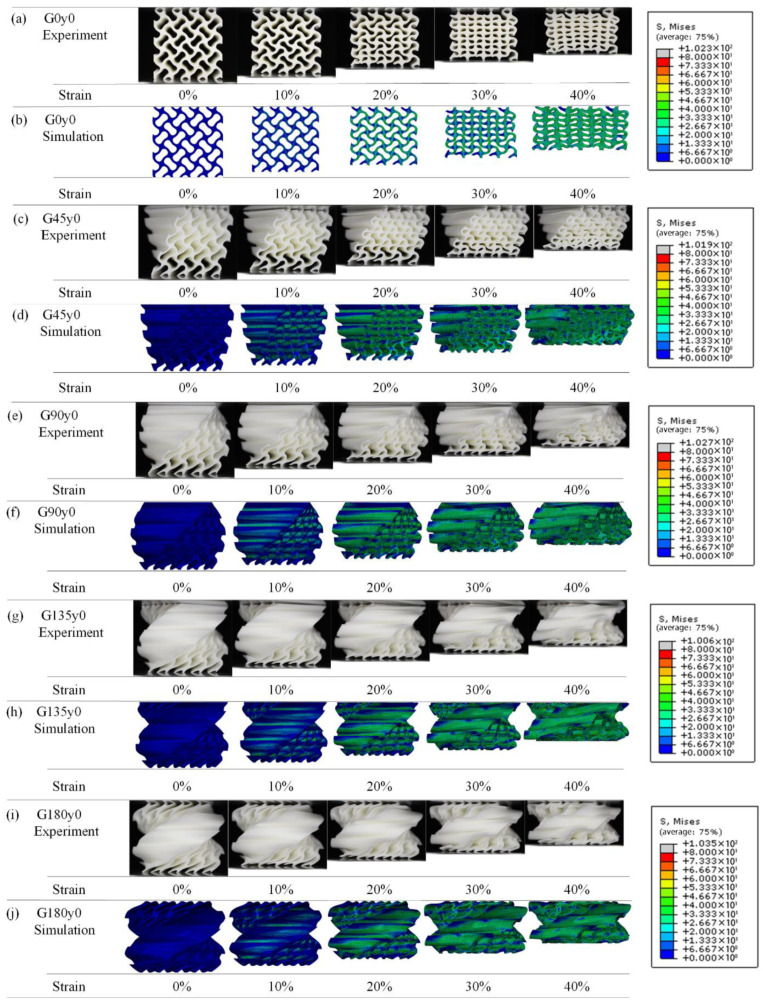
Comparison of experimental and numerical results of Gy0 with different self-twisting angles.

Figure 14 shows the experimental and simulation results of Gy0 structures with different twisting angles. Figure 14a,b show the experimental and simulation results of G0y0, which have been described earlier. Figure 14c,d shows the experimental and simulation results of G45y0. From the simulation results of G45y0, we can see that in the compression process of the structure, the lowest element first has a relatively obvious deformation, and its stress concentration mainly appears at the bottom of the structure, and then gradually transfers upward, which is consistent with the phenomenon in the experimental process. Figure 14e,f show the experimental and simulation results of G90y0. The corresponding simulation results are basically consistent with the experimental results under the same strain. Similar to G45y0, G90y0 also begins to deform from the lowest unit of the structure and bends or even breaks at the bend where the element meets the unit, so the stress is more concentrated at the junction, and each column of elements is inclined and parallel after compression. Figure 14g–j show the experimental and simulation results of G135y0 and G180y0, respectively. It can be seen that the deformation of the finite element simulation model is basically consistent with that of the experiment for the structure with the same twisting angle. With the further increase in the angle, the stress of the structure is still larger at the junction of the element, and there is no obvious stress concentration.

### 3.3. Discussion

**Figure 15:**
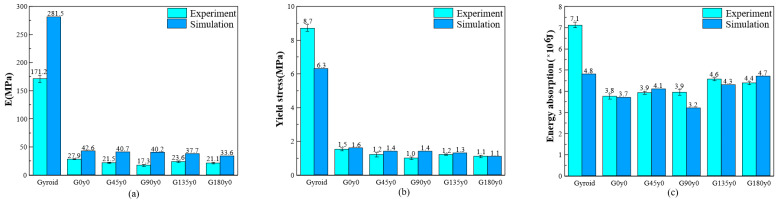
Comparison of mechanical properties between experimental tests and numerical results of the gyroid, G0y0, and other self-twisted lattice structures: (**a**) stiffness; (**b**) yield stress; and (**c**) energy absorption.

Figure 15 compares the experimental and simulation results of the gyroid, G0y0, and other lattice structures with four twisting angles. Figure 15a shows the Young’s modulus diagram based on the slope of different structures in the elastic stage. It can be seen that the simulation results of all structures are larger than the experimental results. Among them, the Young’s modulus of the gyroid structure is the highest in the elastic stage, and the simulation results are 64% higher than the experimental results. The Young’s modulus of the G90y0 structure is the lowest in the elastic stage, and the simulation results are 153.2% higher than the experimental results. From the gyroid to G0y0 structure, the Young’s modulus of the structure decreases greatly. According to the experimental results, the change in the Young’s modulus of the structure is not obvious from 0° to 180°. According to the simulation results, the Young’s modulus of the structure decreases slowly from 0° to 180°, which is basically consistent with the previous analysis. Figure 15b shows the yield stress diagrams of different structures in the experiment and simulation, respectively. Only the simulation results of the yield stress of the gyroid structure are lower than the experimental results, and the simulation results of other structures are greater than or equal to the experimental results. Among them, the yield stress of the gyroid structure is the highest, and the experimental results are 38.1% higher than the simulation results. The yield stress of the G90y0 structure is the lowest, and the simulation results are 40% higher than the experimental results. From the gyroid to G0y0 structure, the yield stress of the structure decreases greatly. According to the experimental results, the change of yield stress of the structure is not obvious from 0° to 180°. According to the simulation results, the yield stress of the structure decreases slowly from 0° to 180°, which is basically consistent with the previous analysis.

Figure 15c shows the energy absorption of different structures in the experiment and simulation. The amount of energy absorption is the area around the stress–strain curve and the strain axis, which reflects the compressive capacity of the structure. As can be seen from the figure, the experimental results of the gyroid, G0y0, G90y0, and G135y0 structures are larger than the simulation results, among which the experimental results of the gyroid structure are the highest compared to the simulation results, which is about 47.9%. The experimental results of G45y0 and G180y0 are smaller than the simulation results, but the difference is not very large. According to the experimental results, the gyroid structure absorbs the most energy and G0y0 absorbs the least energy. From 0° to 180°, the energy absorption shows a gradual upward trend as a whole. According to the simulation results, the gyroid structure absorbs the most energy and G90y0 absorbs the least energy. From 0° to 180°, the energy absorption initially increases, then decreases, followed by an increase. In short, for the Gy0 structure, the gradual increase in the angle has little effect on the Young’s modulus and yield stress in the elastic stage of the structure, but the amount of energy absorption increases gradually with the increase in the twisting angle.

**Figure 16:**
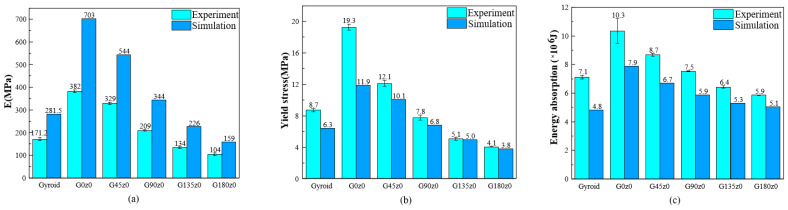
Comparison of mechanical properties between experimental tests and numerical of the gyroid, G0y0, and other self-twisted lattice structures: (**a**) stiffness; (**b**) yield stress; and (**c**) energy absorption.

The experimental and simulation results of the gyroid, G0z0, and other lattice structures with four twisting angles are illustrated in Figure 16. Figure 16a shows the Young’s modulus. As can be seen from the diagram, the Young’s modulus of G0z0 is the highest, and the simulation results are 84% higher than the experimental results. The Young’s modulus of G180z0 is the lowest, and the simulation results are 52.9% higher than the experimental values. From the gyroid to G0z0 structure, the Young’s modulus of the lattice structures is greatly improved. The results show that the Young’s modulus of the structure decreases gradually from 0° to 180°. Figure 16b shows the yield stress from both the experiment and simulation. As can be seen, the yield stress of G0z0 is the highest, and the experimental results are 62.2% higher than the simulation results. The yield stress of the G180z0 structure is the lowest, and the experimental results are 7.8% higher than the simulation results. From the gyroid to G0z0 structure, the yield stress of the structure is greatly increased. The experimental and simulation results show that the yield stress of the structure decreases gradually from 0° to 180°.

The energy absorption of different structures is shown in Figure 16c. The experimental results show that G0z0 absorbs the most energy and G180z0 absorbs the least energy. From 0° to 180°, the energy absorption showed a downward trend. According to the simulation results, G0z0 absorbs the most energy and the gyroid structure absorbs the least energy. Similar to the experimental results, the energy absorption shows a downward trend from 0° to 180°. In summary, for Gz0 structures, the increase in the twisting angle leads to the decrease in the Young’s modulus, yield stress and energy absorption.

**Figure 17:**
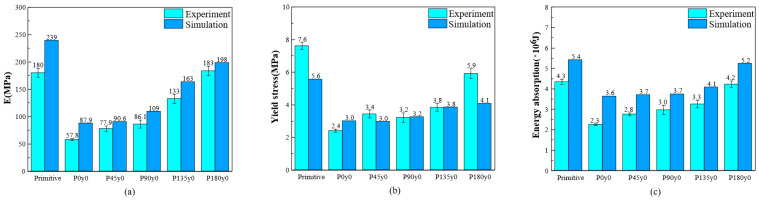
Comparison of mechanical properties between experimental tests and numerical of primitive, P0y0, and other self-twisted lattice structures: (**a**) stiffness; (**b**) yield stress; and (**c**) energy absorption.

The experimental and simulation results of the primitive, P0y0, and other lattice structures with four twisting angles are compared in Figure 17. Figure 17a shows the Young’s modulus. It can be seen that the Young’s modulus of the primitive structure is the highest, and the simulation results are 32.7% higher than the experimental results. The Young’s modulus of P0y0 is the lowest, and the simulation results are 52.1% higher than the experimental results. From the primitive to P0y0 structure, the Young’s modulus of the structure decreases greatly. The results show that the Young’s modulus of the structure increases from 0° to 180°. Figure 17b shows the yield stress of different structures. The primitive structure has the highest yield stress, and the experimental results are 35.7% higher than the simulation results. The yield stress of P0y0 is the lowest, and the experimental results are 25% lower than the simulation results. From the primitive to P0y0 structure, the yield stress of the structure decreases greatly. The results show that the yield stress of the structure increases as a whole from 0° to 180°.

It can be seen from the Figure 17c that both the experimental and simulation results show that the primitive structure absorbs the most energy and P0y0 absorbs the least energy. The energy absorbed by the structure increases from 0° to 180°. In a word, for the Py0 structure, the Young’s modulus, yield stress, and energy absorption of the structure increase with the increase in the twisting angle.

**Figure 18:**
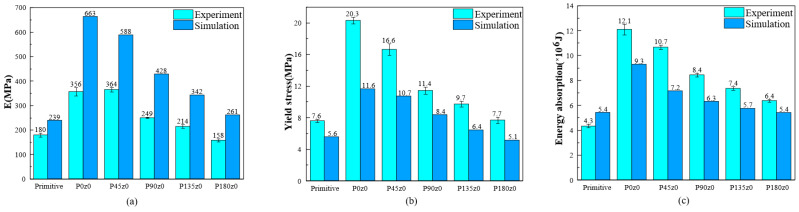
Comparison of mechanical properties between experimental tests and numerical of primitive P0z0 and other self-twisted lattice structures: (**a**) stiffness; (**b**) yield stress; and (**c**) energy absorption.

The experimental and simulation results of the primitive, P0z0, and other lattice structures with four twisting angles are shown in Figure 18. According to the experimental results, the Young’s modulus of P45z0 is the highest and that of P180z0 is the lowest. From the simulation results, the Young’s modulus of P0z0 is the highest and that of the primitive structure is the lowest. From the primitive to P0z0 structure, the Young’s modulus of the structure increases greatly. The results show that the Young’s modulus of the structure decreases as a whole from 0° to 180°. Figure 18b shows the yield stress of different structures. According to the experimental results, the yield stress of P0z0 is the highest and that of the primitive structure is the lowest. Based on the simulation results, the yield stress of P0z0 is the highest and that of P180z0 is the lowest. From the primitive to P0z0 structure, the yield stress of the structure increases greatly. The results show that the yield stress of the structure decreases gradually from 0° to 180°.

The energy absorption of the different structures illustrated in Figure 18c. The experimental results show that P0z0 absorbs the most energy and the primitive structure absorbs the least energy. The energy absorbed by the structure decreases gradually from 0° to 180°. According to the simulation results, P0z0 absorbs the most energy, while P180z0 and the primitive structure absorb the least energy. The energy absorbed by the structure decreases gradually from 0° to 180°. In a word, for the Pz0 structure, the Young’s modulus, yield stress, and energy absorption decrease with the increase in the twisting angle.

It should be noted that there are differences between the simulation results and the experimental results. From the point of view of the experimental sample, this may be due to the internal defect of the 3D-printed sample and the high surface roughness of the sample. From the point of view of the simulation model, this may be the reason for the defined material properties. The printed experimental samples are not as accurate as the finite element simulation model in terms of geometric shape, size, and surface quality, which may lead to various defects, which is a typical issue in additive material manufacturing, and results in the stiffness of the experimental sample being lower than that of the finite element model. In addition, the constitutive material model in the finite element model takes into account the isotropic characteristics, but the selective laser sintering technology may cause the anisotropy of the material properties, resulting in some deviations. In summary, while the finite element model may not precisely replicate the force and deformation of the structure, it still offers valuable insights into the stress distribution within lattice structures.

## 4. Conclusions

In this paper, gyroid and primitive honeycomb lattice structures were designed by fixing one variable in the mathematical expressions of the gyroid and primitive structures, respectively. To improve the controllability of the mechanical properties of lattice structures, the strategy of continuous twisting was proposed, and the self-twisted honeycomb structures with twisting angles of 0°, 45°, 90°, 135°, and 180° were designed to explore the influence of angles on the mechanical performance of honeycomb structures. The mechanical properties of the samples manufactured by selective laser sintering under a quasi-static compression load were experimentally analyzed and numerically investigated. According to the experimental observation and numerical simulation, the following conclusions can be drawn:

(1) The mechanical properties of the honeycomb lattice structure with fixed coordinates are changed largely compared with the original gyroid and primitive lattice structures. Specifically, the Young’s modulus of the honeycomb structures with fixed *y* coordinates (i.e., G0y0 and P0y0) decreases, while the Young’s modulus of the honeycomb structures with fixed *z* coordinates (i.e., G0z0 and P0z0) increases.
(2) The increase in the twisting angle has no significant effect on the elastic stage of the honeycomb structure with fixed *y* coordinates, but it can increase the energy absorption of the structure, shorten the platform stage, and make the structure enter the densification stage faster. For the honeycomb structure with fixed *z* coordinates, with the gradual increase in the angle, the Young’s modulus and the maximum stress peak value of the structure in the elastic stage gradually decrease, but the collapse of the structure is gradually alleviated, which makes the structure enter the platform stage more smoothly.
(3) A finite element model is also developed to evaluate the stress distribution of each lattice structure under different strains and to analyze the deformation mechanism of some key areas of each structure. It can be noted that the numerical results are consistent with the experimental observations and help to understand the compression process.
(4) Both numerical and experimental results show that twisting has an important influence on the mechanical properties in different directions. Although the twisting may lead to the decrease in the Young’s modulus of the structure, it also plays a role in the collapse and fracture of the lattice structures. Overall, the results obtained show that a twisting design has great potential in creating lattice structures with largely tunable mechanical properties.

## Data Availability

Data are contained within the article.
